# The effect of water level in vertical aquaculture systems on production performance, biochemistry, hematology, and histology of *Anguilla bicolor bicolor*

**DOI:** 10.1038/s41598-021-90912-1

**Published:** 2021-05-31

**Authors:** Eko Harianto, Eddy Supriyono, Tatag Budiardi, Ridwan Affandi, Yani Hadiroseyani

**Affiliations:** 1 Doctoral Program Student, Graduate School, Department of Aquaculture, Faculty of Fisheries and Marine Sciences , IPB University , Jalan Agatis, IPB Dramaga , Bogor, 16680 Indonesia; 2 Aquaculture Study Program, Faculty of Agriculture, Jambi Batanghari University , Jl. Slamet Ryadi, Broni Jambi, 36122 Indonesia; 3 Department of Aquaculture, Faculty of Fisheries and Marine Sciences, IPB University , Jalan Agatis, IPB Dramaga , Bogor, 16680 Indonesia; 4 Department of Aquatic Resources Management, Faculty of Fisheries and Marine Sciences, IPB University , Jalan Agatis, IPB Dramaga , Bogor, 16680 Indonesia

**Keywords:** Biochemistry, Biological techniques

## Abstract

The water level in the cultivation of eel (*Anguilla bicolor bicolor*) is an important study in order to provide the optimal water level for cultivation. Optimizing the water level will affect the substitution of respiration energy with energy to grow. In addition, the water level information is related to the efficiency of water use for eel production in the future. Information on water level for eel production is still very limited, so this research is necessary to do. A total of 120 eel elver (initial weight 13.66 ± 0.09 g) were collected from eel companies in Bogor City, Indonesia. Fish were reared in vertical aquaculture systems with a stocking density of 10 fish per container for 60 days. The artificial feed containing 55% protein given as much as 3–5% of the biomass. Absorption and water replacement were done 20% per day. The result of this research showed that fish weight increased with an average of 33.45 ± 0.33 g. Different water levels had an impact to KKb, SGOT, ALP, and He. There was erosion of the skin epidermis and necrosis of the gill filaments due to the adaptation process. Water quality was within the optimum range for all treatments and 1.5 cm water level is recommended for maintenance (SGOT, ALP and He values were closest to normal values).

## Introduction

*Anguilla* spp. eel is a catadromous fish that lives in two habitats, namely marine waters during the larval stage and inland waters (brackish and fresh) during the juvenile to adult stage^1,2^. A total of 19 eel species exist in tropical and sub-tropical waters^3^. Thirteen species inhabit tropical and six species are in subtropical waters. One type of tropical eels is *Anguilla bicolor bicolor* which is found on the west coast of Sumatra Island and the south coast of Java Island^4,5^ As with eels in general, *A. bicolor bicolor* eels also carry out migration in their life cycle. During this migration phase, eels are sometimes encounter extreme water condition such as changes in water levels^1^. This occurs when eels migrate upstream (fresh water) through limited water and semi-dry rivers^6^. This condition requires eels to adapt to water level changes. The study of the migration physiology of eels has been reported, especially for captured eels which have been analyzed for their physiological responses^7–9^.

In contrast to cultivated eels, slight information is available regarding the evaluation of water levels for the aquaculture process. This is because the use of volume and water depth is still high in the cultivation process. The breeding and rearing process of laboratory-scale eels with a regular aquarium using water depth and volume ranged from 20 to 35 cm^10–13^. Meanwhile, for the production scale with pond, the water level commonly used is between 50 and 200 cm^14–16^. The water level in eel fish farming will have a direct impact on physiological responses, growth and survival. The water level will determine the amount of energy used by the fish to absorb oxygen at the surface and reduce the energy utilization for growth. The *Anguilla anguilla* eels spend a lot of energy in vertical migration so their acceleration is slower and the distance covered is shorter^17^. During the cultivation process, if the dissolved oxygen in the rearing container < 1 mg L^−1^, the eels will swim to the surface of the water, the position of the body is in direct contact with free air and the head is hidden out of the water with the gill cavities that swell to resemble a vertical pipe^18,19^. This swimming behavior will last a long time and cause the fish to run out of energy which can lead to death so that specific information is needed regarding the optimal water level for eel cultivation.

Optimizing water use is related to efficiency. The low water level with a small volume will result in high water use efficiency in producing fish biomass. Eel elver *A. bicolor bicolor* stadia are able to live and grow at a water level of 1.5 cm or as high as its body, but the results of this study still showed low production performance^20^. The growth of *A. bicolor bicolor* eel increased with the reduction of water volume^21,22^. In addition, eel farming commonly still employs horizontal land, so that the efficiency of land usage is still low. The vertical cultivation is a system that makes use of land vertically. This system has been widely developed for crop production^23–26^ and some aquatic organisms such as the spiny lobster *Jasus edwardsii*^27^ and mud crab^28^. Information on the vertical eel cultivation system has not been extensively reported thus it is a great opportunity to improve this system in the context of efficient water and land utilization for eel cultivation in the future. This study aims to evaluate the effect of water level on the maintenance of eel elver *A. bicolor bicolor* with vertical aquaculture system (VAS) on physiological conditions and production performance.

## Material and methods

This research was conducted from June to August 2019 at the Laboratory of Production Technology and Aquaculture Management, Aquaculture department, Faculty of Fisheries and Marine Science, IPB University. The analysis of blood biochemistry was carried out at the animal teaching hospital, Faculty of Veterinary Medicine, IPB University. The analysis of hematology, histology, and water quality was conducted in a laboratory within the Aquaculture department, Faculty of Fisheries and Marine Science, IPB University. This study has met the guidelines and protocols approved by the Animal Ethics Committee of IPB University. This research was also accompanied by the staff of the Ethics Committee for the stages of using test animals, anesthetizing test animals, and taking blood samples. The authors of this research complied with the ARRIVE guidelines.

### Experimental animals and container set up

**Figure 1:**
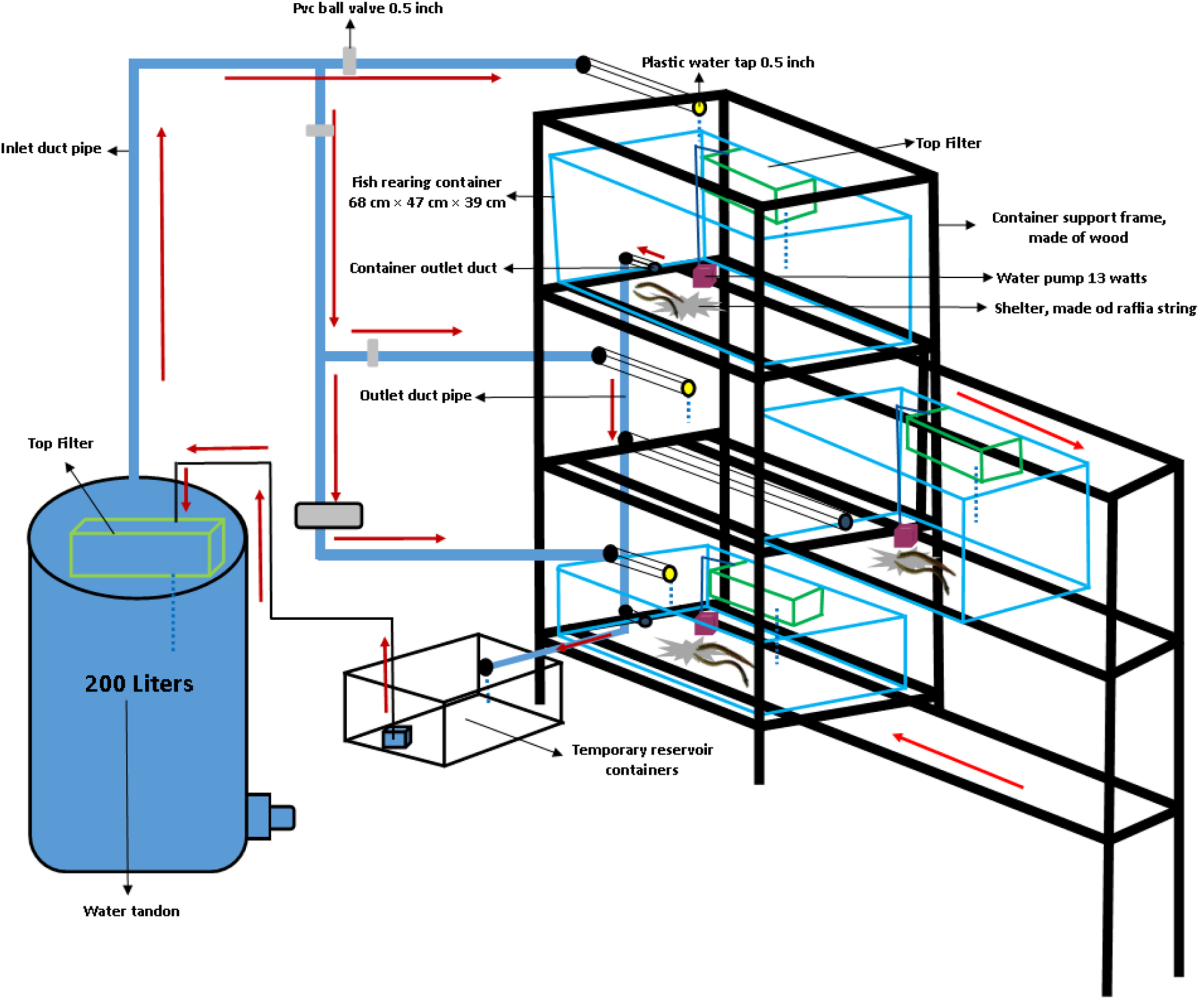
Experimental set up, vertical aquaculture systems (the container can be stretched horizontally to the front).

The eel elver in this study (average weight 13.66 ± 0.09 g) is a fish cultivated from an eel company in Bogor City, West Java, Indonesia. A total of 120 fish were stocked into 12 rearing containers (68 cm × 47 cm × 39 cm), arranged vertically (10 fish per container^19^), and reared for 60 days. The experiments were divided into four groups. Among the twelve units of eel rearing containers, different water levels were given for each of the three rearing containers which included a water level of 1.5 cm (A), 2.25 cm (B), 3.0 cm (C) and 3.75 cm (D). The vertical aquaculture system constructed for rearing the experimental fish was designed by arranging three cultivation tubs vertically like a drawer with wooden support frames, each was equipped with a recirculation system (Fig. 1). The maintenance container measuring 68 cm × 47 cm × 39 cm, each of which was supplied with supporting components such as a top filter containing synthetic cotton, activated carbon and zeolite, a 13-W water pump with a total discharge of 0.07 L s^−1^ and aeration that functioned to supply oxygen as well as shelter for the test fish made of raffia strings. The water reservoir in this experiment was only used in the water change process. The water from reservoir was flowed through a 0.5-in. PVC pipe to the cultivation tubs. The water flow that entered the maintenance container was adjusted by using a water tap according to the percentage of water change. Water from the maintenance tubs came out through the outlet channel in the form of a 1 cm aeration hose located on the bottom wall of the tub. This outlet channel functioned to control the water level during the water change process. The water then was streamed into a temporary storage tank by gravity which is then flowed back to the reservoir using a pump.

### Feed and water preparation

During the experimental process, the fish were fed three times a day with limited feed amount of 3–5% from the biomass in each rearing container. The type of feed given was commercial powder (PIS 1-Japfa PT Suri Tani Pemuka, 55% crude protein, 5% crude fat, 18% crude ash, 2% fiber and 11% moisture content). The feed was reformed into a paste or dough by mixing it with water (the ratio is1 g feed: 0.25 ml water). Feed making was carried out every sampling period by adjusting the fish biomass in the rearing container. Water management in the container was performed by siphonize and changing the water. The waste of feed eliminated by siphoned off before feeding and water change (as much as 20% of water volume in the container)^10^ is done twice a day (morning and evening).

### Sampling of fish, blood and water

The data in this study were collected through weight, blood, water sampling on every 15 days during the maintenance period, while gills and liver sampling were collected before and after the maintenance period. Fish were removed from the maintenance container and placed in a sampling box, and then measured individually using the digital scale at 0.01 g accuracy. The blood sample was taken from the fish after being sedated with a stabilizer (1 mL/2 L). Sample of blood was taken from the upper side between anus and anal fin end for hematology and blood chemistry analysis. Blood hematology sample was collected as much as 0.5 mL by a syringe filled with an anticoagulant (sodium-citrate) and distributed into the Eppendorf tube based on the treatment groups. Blood biochemistry sample was obtained as much as 1.5 mL without coagulant and distributed in the EDTA tube based on the treatment groups. Blood sampling was carried out for 10–15 min. The first blood sample was used for the hematological analysis and second blood sample was used for the blood biochemical analysis. Water sample was collected taking 250 mL water from the maintenance media and analyzed in the laboratory. The skin and gill samples were collected from the oversedated fish and cut in the head base to obtain the gill organs, while the back part was vertically cut to obtain the skin using scissors and tweezer.

### Measurement of production performance

The standard formulas used to determine the survival rate, SR (%) = (N_t_/N_0_) × 100, whereas N_t_ was the total final fish (fish) and N_0_ was the total initial fish (fish). Weight specific growth rate, wSGR (%) = ((ln w_t_ – ln w_0_)/t) × 100, whereas t was the maintenance period = 60 days, w_t_ = the average individual weight at 60 days (g) and w_0_ = the average individual weight at 0 day (g), absolute growth rate, AGR (g/day) = (w_t_ – w_0_)/t, biomass absolute growth rate, bAGR (g/day) = (B_t_ – B_0_)/t, whereas B_t_ = biomass at 60 days (g), B_0_ = biomass at 0 day (g), feed conversion, FCR = consumed feed (g)/increased weight (g), weight diversity coefficient at 60 days, wDC (%) = (100 × SDW)/W, whereas W was the average individual weight at 60 days (g) and SDW was the weight standard deviation, oxygen consumption rate, OCR (mgO_2_ /g/h) = V × (DO_0_ – DO_t_)/(w × t), whereas V = water volume in the container (L), DO_0_ = dissolved oxygen at 0th period (mg/L), DO_t_ = dissolved oxygen at the *t*th period (mg/L), w = fish sample weight (g). The OCR was measured by preparing the 5 L closed container with no air space remained and strong aeration for 24 h. The aeration was removed, DO_0_ was measured and noted after the aeration was removed. Fish sample was measured and moved into the container, and then closed and measured the Dot each hour for 3 h ^29^.

### Blood biochemical analysis

The blood chemical parameters contained blood glucose (BG), total protein (TP), serum glutamic pyruvic transaminase (SGPT), serum glutamic oxaloacetic transaminase (SGOT), alkaline phosphatase (ALP), blood urea nitrogen (BUN) and creatinine. The analysis was performed using the blood chemical analyzer SPOTCHEM-EZ sp 4430 (ARKRAY Inc., Kyoto, Japan) equipped with the paper test indicator for each parameter. The blood biochemical analysis was performed in the Animal Hospital, Faculty of Animal Medicine, IPB University.

### Hematological analysis

The total red blood cell (RBC) was calculated following^30^ by absorbing the blood sample with an absorbent pipette filled with red colored pulp stirrer until reaching scale 1, and then added with the Hayem’s solution until reaching scale 101, shaken for 3–5 min until homogenous. The first drop was removed and the blood was dropped into the hemacytometer enclosed with the cover glass and observed under the microscope. The calculation was performed on the small box of hemacytometer with the following formula: ∑ RBC = ∑ counted RBC × 10^6^ cells/mm^3^.

The total white blood cell (WBC) was calculated following^30^ by absorbing the blood sample with an absorbent pipette filled with white colored pulp stirrer until reaching 0.5 mL scale, and then added with the Turk’s solution until reaching scale 11, shaken for 5 min until homogenous. The first drop was removed and the blood was dropped into the hemacytometer enclosed with the cover glass and observed under the microscope. The calculation was performed on the small box of hemacytometer with the following formula: ∑ WBC = ∑ counted WBC × 10^4^ cells/mm.

The hemoglobin (Hb) measurement was performed following the Sahli method with the Sahlinometer/Hb meter^31^. Blood was absorbed with the Sahli pipette until reaching 20 mm^3^ or 0.2 mL scales, then the pipette end was cleaned with tissue. Blood in the pipette was moved into the Hb-meter tube filled with 0.1 N HCl until reaching scale 10 (red), then stirred and stood for 3–5 min. Aquadest was added into the blood sample and HCl until the color was similar to the Hb meter. The scale was read by observing the liquid surface and matched with the Sahli tube in the line scale of gr% (yellow), which presented the hemoglobin content in gram per 100 ml blood.

The hematocrit (He) was calculated based on^32^. The blood sample was moved into the microhematocrit tube approximately reached 3/4 tube part, then the end of the tube was plugged with the 1 mm crystoseal and centrifuged at 5000 rpm for 5 min. The precipitated blood was measured its length (a) and total blood volume in the tube (b) using a ruler. The HE content was presented in % solid blood cell volume and calculated following the formula: HE = (a/b) × 100%.

The leukocyte differentiation (LD) was calculated based on^30^. Blood was dropped on the methanol-soaked sterile object glass and the end of the second object glass was placed on the blood dropped object glass until forming 30° angle. The second object glass was moved to the back, while touching the blood drop until the blood spreaded and was stood until dry. The spreaded blood was fixated with the methanol absolute for 5 min, then removed and stood until dry. The blood sample staining was performed for 10 min in the Giemsa solution, then removed and rinsed with the flowing water, stood until dry. The blood smear was observed under the microscope at 1000× magnification. The leukocyte types were calculated their percentage from 100 leukocytes in several observational field. The hematological analysis was performed in the Fish Health Laboratory, Faculty of Fisheries and Marine Sciences, IPB University.

### Histological analysis

The histological observation of gill and skin organs was performed following method^33^ by fixating the organs with Bouin’s solution and dehydrated into the serial alcohol from 70% until absolute, then infiltrated and blocker into the liquid paraffin. The histological samples were stained with the hematoxylin and eosin (HE), then observed under the microscope at 40× magnification. The analysis results in the form of figures were added with 50 μm scale bar. The histological analysis was performed in the Fish Health Laboratory, Faculty of Fisheries and Marine Sciences, IPB University.

### Water quality analysis

The water quality parameters containing temperature, pH, and dissolved oxygen (DO) were directly measured every day using the mercury thermometer, Hanna HI98107 pH meter, and Lutron-5510 DO meter. TAN, ammonia, nitrite and alkalinity were analyzed using the spectrophotometer and Titimetrical method^34,35^. The water quality analysis was performed in Environmental laboratory, Faculty of Fisheries and Marine Sciences, IPB University.

### Statistical analysis

The data obtained were tabulated with Microsoft Office Excel 2013 (Microsoft Corp., Washington, USA) and SPSS 22.0 (IBM Corp., New York, USA) for analysis of variance to determine the treatment effect in each parameter at 95% confidence level. Duncan test was performed when there were significant differences among treatment groups based on the parameters obtained.

## Results

### Production performance

Table 1 summarized production performance of eel elver after 60 days rearing period. Different water level treatments had no significant effect (*p *> 0.05) on the parameters of SR, WSGR, AGR, AGRoB, FCR, and OCR. Meanwhile, the results showed that the treatment had a significant effect (*p *< 0.05) on WCD. The highest value of WCD was indicated in treatment B of with 11.41%. Relatively similar results were shown in treatment A and C with the values of 8.87% and 10.01%, respectively. The lowest WCD was found in treatment D with 7.36%.

**Table 1:** Production performance of *A. bicolor bicolor* reared for sixty days at several different water levels.

Item	Water level				
	r	A (1.5 cm)	B (2.25 cm)	C (3 cm)	D (3.75 cm)
SR (%)	1	100	100	100	100
	2	100	100	100	100
	3	100	100	100	100
Average ± SD		100.00 ± 0.00	100.00 ± 0.00	100.00 ± 0.00	100.00 ± 0.00
WSGR (%)	1	1.38	1.55	1.52	1.47
	2	1.50	1.49	1.46	1.57
	3	1.57	1.50	1.54	1.50
Average ± SD		1.48 ± 0.09^a^	1.51 ± 0.03^a^	1.50 ± 0.04^a^	1.51 ± 0.05^a^
AGR (g/day)	1	0.29	0.35	0.34	0.32
	2	0.33	0.33	0.32	0.35
	3	0.34	0.32	0.34	0.34
Average ± SD		0.32 ± 0.03^a^	0.33 ± 0.02^a^	0.33 ± 0.01^a^	0.33 ± 0.02^a^
AGRoB (g/day)	1	2.42	2.93	2.82	2.62
	2	3.31	3.25	3.21	3.51
	3	3.44	3.16	3.36	3.37
Average ± SD		3.05 ± 0.05^a^	3.11 ± 0.17^a^	3.13 ± 0.28^a^	3.16 ± 0.48^a^
FCR	1	1.87	1.65	1.72	1.82
	2	1.49	1.52	1.60	1.51
	3	1.43	1.56	1.48	1.48
Average ± SD		1.60 ± 0.24^a^	1.58 ± 0.06^a^	1.60 ± 0.12^a^	1.60 ± 0.19^a^
WCD (%)	1	7.72	13.12	10.71	8.16
	2	10.03	12.09	10.51	7.65
	3	8.87	9.04	8.81	6.28
Average ± SD		8.87 ± 1.16^ab^	11.41 ± 2.12^b^	10.01 ± 1.04^ab^	7.36 ± 0.97^a^
OCR (mgO_2_/g/h)	1	0.21	0.19	0.20	0.20
	2	0.22	0.17	0.21	0.16
	3	0.19	0.21	0.19	0.19
Average ± SD		0.21 ± 0.02^a^	0.19 ± 0.02^a^	0.20 ± 0.01^a^	0.19 ± 0.02^a^

Data are presented as mean ± SEM. The values with same letters in the same line indicate non-significant differences (*p* > 0.05) in 5% significance level. One-way analysis of variance (ANOVA) followed by Duncan’s multiple-range test was used to test significant differences among groups.

*r *repetition, *SR* survival rate, *WSGR *weight-specific growth rate, *AGR *absolute growth rate, *AGRoB *absolute growth rate of biomass, *FCR *feed conversion ratio, *WCD* weight coefficient diversity, *OCR *oxygen consumption rate.

### Blood biochemistry

Different water level treatments had no significant effect (*p* > 0.05) on the parameters of BG, TP, SGPT, BUN, and creatinine. Meanwhile, the treatments had a significant effect (*p* < 0.05) on both SGOT and ALP (Table 2). The highest SGOT value was found in treatment A with 128.44 IU/L. Treatment C and D shared somewhat similar results with 113.56 IU/L and 111.22 IU/L, respectively. The lowest SGOT was found in treatment B with 100.11 IU/L. The SGOT value decreased as compared to that before the treatments that showed 129.00 IU/L. Meanwhile, the highest ALP value was found in treatment C with 288.89 IU/L. The lowest ALP value was shown in treatment A, which was 249.33 IU/L. The values of treatment B and D were 267.44 IU/L and 225.72 IU/L, respectively. The ALP value before the treatments was 265.00 IU/L. The value indicated a decrease in treatment A and an increase in all other treatments.

**Table 2:** Blood biochemistry parameters of A*. bicolor bicolor* reared for sixty days at several different water levels.

Item	Water level					
	r	Before treatment	A (1.5 cm)	B (2.25 cm)	C (3 cm)	D (3.75 cm)
BG (mg/dL)	1	61 ± 22.52	25.75	37.25	38.25	41.75
	2		32.75	23.25	21.75	37.75
	3		27.25	18.75	36	21
Average ± SD			28.58 ± 3.69^a^	26.42 ± 9.65^a^	32.00 ± 8.95^a^	33.50 ± 11.01^a^
TP (g/dL)	1	4.00 ± 0.00	3.17	3.50	3.43	3.50
	2		3.53	3.23	3.87	3.80
	3		3.40	3.43	3.60	3.60
Average ± SD			3.37 ± 0.19^a^	3.39 ± 0.14^a^	3.63 ± 0.22^a^	3.63 ± 0.15^a^
SGPT (IU/L)	1	3.00 ± 0.00	1.00	1.00	1.00	1.00
	2		1.00	1.00	1.00	1.00
	3		1.00	1.00	1.00	1.00
Average ± SD			1.00 ± 0.00^a^	1.00 ± 0.00^a^	1.00 ± 0.00^a^	1.00 ± 0.00^a^
SGOT (IU/L)	1	129.00 ± 0.00	115.33	109.00	118.33	122.00
	2		133.33	98.33	114.00	100.33
	3		136.67	93.00	108.33	111.33
Average ± SD			128.44 ± 11.48^a^	100.11 ± 8.15^b^	113.56 ± 5.01^ab^	111.22 ± 10.83^ab^
ALP (IU/L)	1	265 ± 0.00	241.00	263.00	275.67	216.50
	2		238.00	268.00	313.50	231.67
	3		269.00	271.33	277.50	229.00
Average ± SD			249.33 ± 17.10^ab^	267.44 ± 4.19^bc^	288.89 ± 21.33^c^	225.72 ± 8.10^a^
BUN (mg/dL)	1	5.00 ± 0.00	5.00	5.00	5.00	5.00
	2		5.00	5.00	5.00	5.00
	3		5.00	5.00	5.00	5.00
Average ± SD			5.00 ± 0.00^a^	5.00 ± 0.00^a^	5.00 ± 0.00^a^	5.00 ± 0.00^a^
Creatinine (mg/dL)	1	0.90 ± 0.00	0.37	0.40	0.37	0.40
	2		0.43	0.40	0.40	0.40
	3		0.40	0.40	0.40	0.37
Average ± SD			0.40 ± 0.03^a^	0.40 ± 0.00^a^	0.39 ± 0.02^a^	0.39 ± 0.02^a^

Data are presented as mean ± SEM. No statistical analyses were performed on fish before treatment. The values with same letters in the same line indicate non-significant differences (*p* > 0.05) in 5% significance level. One-way analysis of variance (ANOVA) followed by Duncan’s multiple-range test was used to test significant differences among groups.

*r* repetition, *BG* blood glucose, *TP* total protein, *SGPT* serum glutamic pyruvic transaminase, *SGOT* serum glutamic oxaloacetic transaminase, *ALP* alkaline phosphatase, *BUN* blood urea nitrogen.

### Hematology

The hematology is presented by several indicators, including RBC, WBC, Hb, He, and DL (monocytes, lymphocytes, and neutrophils) (Table 3). The treatments of different water levels had a significant effect (*p* < 0.05) on the He value with the highest value found in treatment D with 27.31%. This value was the same as that of treatment B, but different from those of treatment A and C. The lowest He value was found in treatment C with 25.20%. In general, the He value increased by 21.60% as compared to that before the treatment.

**Table 3:** Hematology parameters of A*. bicolor bicolor* reared for sixty days at several different water.

Item	Water level					
	r	Before treatment	A (1.5 cm)	B (2.25 cm)	C (3 cm)	D (3.75 cm)
RBCs(× 10^6^ sel/mm^3^)	1	1.27 ± 0.00	1,490,000	1,680,000	1,370,000	1,470,000
	2		1,378,000	1,608,000	1,408,000	1,520,000
	3		1,570,000	1,504,000	1,592,000	1,558,000
Average ± SD			1.48 ± 0.10^a^	1.60 ± 0.09^a^	1.46 ± 0.12^a^	1.52 ± 0.04^a^
WBCs(× 10^4^ sel/mm^3^)	1	9.27 ± 0.00	85,520	70,040	73,600	52,000
	2		60,540	82,200	71,680	76,360
	3		89,080	78,500	79,400	83,420
			15,552.09	6233.34	4019.22	16,484.69
Average ± SD			7.84 ± 1.56^a^	7.69 ± 0.62^a^	7.49 ± 0.40^a^	7.06 ± 1.65^a^
Hb (gram %)	1	7.20 ± 0.00	8.52	7.64	8.8	8
	2		8.4	8.9	9.34	8.72
	3		8.44	8.94	8.72	8.86
Average ± SD			8.45 ± 0.06^a^	8.49 ± 0.74^a^	8.95 ± 0.34^a^	8.53 ± 0.46^a^
He (%)	1	21.60 ± 0.00	26.26	27.4	24.24	26.6
	2		25.16	27.28	25.12	28.94
	3		26	28.34	26.24	26.4
Average ± SD			25.81 ± 0.57^a^	27.67 ± 0.58^b^	25.20 ± 1.00^a^	27.31 ± 1.41^b^
Monocytes (%)	1	25.20 ± 0.00	10.18	13.24	14.32	10.76
	2		10.8	15.2	13.9	20.28
	3		10.76	10.22	9.92	12.72
Average ± SD			10.58 ± 0.35^a^	12.89 ± 2.51^a^	12.71 ± 2.43^a^	14.59 ± 5.03^a^
Lymphocytes (%)	1	61.80 ± 0.00	74.56	72.84	72.88	76.66
	2		69.48	70.68	71.6	66.2
	3		75.44	74.16	76.38	71.24
Average ± SD			73.16 ± 3.22^a^	72.56 ± 1.76^a^	73.62 ± 2.47^a^	71.37 ± 5.23^a^
Neutrophils(%)	1	13.00 ± 0.00	15.26	14.5	12.8	12.58
	2		19.76	14.1	14.48	13.52
	3		13.78	15.62	13.7	16.04
Average ± SD			16.27 ± 3.11^a^	14.74 ± 0.79^a^	13.66 ± 0.84^a^	14.05 ± 1.79^a^

Data are presented as mean ± SEM. No statistical analyses were performed on fish before treatment. The values with same letters in the same line indicate non-significant differences (*p* > 0.05) in 5% significance level. One-way analysis of variance (ANOVA) followed by Duncan’s multiple-range test was used to test significant differences among groups.

*r* repetition, *RBCs* red blood cells, *WBCs* white blood cells, *Hb* hemoglobin, *He* hematocrit.

### Gill and skin histology

**Figure 2:**
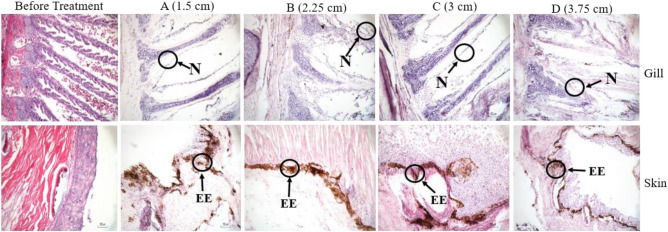
Histology of gill and skin *A. bicolor bicolor* reared for sixty days at several different water levels. N = necrosis, EE = erosion of epidermis, Hematoxylin–Eosin 40× (f) bar = 50 μm.

The gill and skin histology at different water levels is shown in Fig. 2. The results showed that different water levels caused damages to the gills and skin. For instance, gill filament necrosis occurred in all treatments. The skin also experienced some changes in its histological structure, namely erosion, and irritation of its epidermis in all treatments.

### Water quality

The water quality of eel elver rearing for 60 days showed that treatment had no significant effect on pH, TAN, nitrite, ammonia, and alkalinity but had effect on temperature and DO (Table 4). In general, the water quality of rearing process was still in a proper condition for the cultivation of eel elver. The highest temperature was found in treatment A (28.48 °C) and the lowest was in treatment D (27.01 °C). The highest DO concentration was identified from treatment A (5.28 mg/L) and the lowest was from treatment B (4.90 mg/L). The concentration of pH ranged from 6.88 to 6.92; concentration of TAN ranged from 0.42 to 0.51 mg/L; concentration of nitrite ranged from 0.84 to 1.58 mg/L; concentration of ammonia ranged from 0.0024 to 0.0038 mg/L; and concentration of alkalinity ranged from 13.42 to 16.42 mg/L.

**Table 4:** Water quality parameters of *A. bicolor bicolor* reared for sixty days at several different water levels.

Water quality	Treatment (water level)					
	r	A (1.5 cm)	B (2.25 cm)	C (3 cm)	D (3.75 cm)	Optimum value (references)
Temperature (℃)	1	28.82	28.82	27.81	27.02	23–32^95^
	2	27.81	27.81	28.78	27.02	
	3	28.82	27.81	26.98	26.98	
Average ± SD	28.48 ± 0.58^b^	28.15 ± 0.58^ab^	27.86 ± 0.90 ^ab^	27.01 ± 0.03^a^		
pH	1	6.91	6.88	6.89	6.91	6–8^96^
	2	6.91	6.88	6.92	6.89	
	3	6.85	6.90	6.94	6.84	
Average ± SD	6.89 ± 0.03^a^	6.89 ± 0.01^a^	6.92 ± 0.02^a^	6.88 ± 0.04^a^		
DO (mg/L)	1	5.35	5.04	4.91	5.21	> 3.0^98^
	2	5.39	5.00	5.15	5.32	
	3	5.09	4.67	4.94	4.92	
Average ± SD	5.28 ± 0.16^b^	4.90 ± 0.20^ab^	5.00 ± 0.13^ab^	5.15 ± 0.21^a^		
TAN (mg/L)	1	0.72	0.40	0.51	0.44	< 0.25^96^
	2	0.38	0.41	0.43	0.43	
	3	0.43	0.52	0.34	0.39	
Average ± SD	0.51 ± 0.19^a^	0.44 ± 0.07^a^	0.42 ± 0.09^a^	0.42 ± 0.03^a^		
Nitrite (mg/L)	1	0.94	0.99	1.90	1.96	< 0.5^99^
	2	0.46	1.03	1.30	1.05	
	3	1.13	1.87	1.31	1.74	
Average ± SD	0.84 ± 0.34^a^	1.30 ± 0.50^a^	1.50 ± 0.34^a^	1.58 ± 0.47^a^		
Ammonia (mg/L)	1	0.0044	0.0035	0.0045	0.0024	< 0.1^94^
	2	0.0022	0.0038	0.0026	0.0028	
	3	0.0035	0.0033	0.0042	0.0021	
Average ± SD	0.0034 ± 0.0011^a^	0.0035 ± 0.0003^a^	0.0038 ± 0.0010^a^	0.0024 ± 0.0003^a^		
Alkalinity (mg/L)	1	11.50	20.00	15.50	15.75	30–500^102,103^
	2	13.00	14.25	15.50	15.00	
	3	15.75	14.75	18.25	18.25	
Average ± SD	13.42 ± 2.16^a^	16.33 ± 3.19^a^	16.42 ± 1.59^a^	16.33 ± 1.70^a^		

Data are presented as mean ± SEM. The values with same letters in the same line indicate non-significant differences (*p* > 0.05) in 5% significance level. One-way analysis of variance (ANOVA) followed by Duncan’s multiple-range test was used to test significant differences among groups.

*r* repetition, ^95^Ritonga (2014), ^96^Tseng and Wu (2004), ^98^Herianti (2005), ^99^Knosche (1994), ^94^KKP (2011), ^102^Wahyudi et al. (2015); ^103^Chaudhary and Pillai (2008).

## Discussion

The production performance is a major parameter in the production of aquaculture biota that can indicate production success^36^. Based on Table 1, it can be seen that the production performance of the eel *A. bicolor bicolor* stadia elver in all treatments showed a significant increase. The SR in all treatments was 100% indicating that no death was found during the study. The values of WSGR, AGR, and AGRoB ranged from 1.48 to 1.51%, 0.32 to 0.33 g/day, and 3.05 to 3.16 g/day, respectively. FCR and OCR ranged from 1.58 to 1.60 and 0.19 to 0.21 mgO_2_/g/h, respectively. In general, this study shows better results than the previous studies about the use of water volume of culture media, individual growth rate, and biomass of the eel *A. bicolor bicolor* stadia elver reared for seventy days at a water volume of 0.4 m^3^ resulting in SR values of 97.98–98.54%, WSGR 0.72%, and FCR 1–1.72^22^. Meanwhile, the eel *A. bicolor bicolor* stadia elver with a size of 20 g/e reared at a water level of 1.5 cm or as high as its body produced SR values of 66.67–100%, WSGR 0.17–0.45%, AGRoB 0.02–0.07 g/day, and FCR 6.57–23.08^20^. The results of this study are also better when compared to the cultivation of *A. bicolor bicolor* eels in the aquaponic system and phytoremediation using high water volume, the resulting SR value of 78–100%, the growth was 0.3–1.19% and the KP was 1.57–3.91^13,37^. Besides, the growth in this study is higher than that of the eel *A. bicolor bicolor* stadia elver reared in a water volume of 48–120 L indicating the values between 0.2 and 1.47% for its WSGR^10,11,13,38^ and 0.8–3.48 g/day for its AGR^12,38^.

In the European eel *A. Anguilla* reared in different sizes, the results of WSGR and FC were 0.48–0.6% and 1.8–2.2, respectively^39^. Meanwhile, WCD illustrates the level of weight diversity at the end of the rearing period. The higher the WCD value, the smaller the level of weight uniformity will be. The highest WCD in this study was found in treatment B with 11.41%; this value was relatively similar to the values of treatment A and C, but different from the result of treatment D that showed only 7.36% of WCD. The WCD values in this study were still below 20%, indicating that the weight uniformity rate of the eel *A. bicolor bicolor* stadia elver at the end of the study was high^40^. This study has better results of the WCD values than the previous studies where the eels were reared in a water volume of 48–120 L with the WCD value ranging from 12 to 39%^10–12^. OCR functions as an indicator to determine the metabolic rate of fish^41^. The lower the TKO rate, the less energy used for metabolism that more energy available for growth. The results showed that OCR ranged from 0.19 to 0.20 mgO_2_/g/h. The values are lower than those of the previous studies with 0.26–0.4 mgO_2_/g/h of OCR^42,43^.

In general, the water level treatment in this study did not show any significant effect (*p* < 0.05) on the production performance parameters. It implies that the water level in the treatment is still within normal limits to support SR and the growth of the fish. In terms of water use efficiency, the lowest water level is the most efficient treatment in using water for the cultivation of the eel *A. bicolor bicolor.*

Blood biochemistry is a secondary response of fish to environmental changes and pathogenic infections that cause fish to be in a stressful state due to the release of several stress hormones^44^. This secondary response is also characterized by changes in blood and tissue biochemistry, such as the increase of blood glucose^45,46^. It eventually results in a large amount of energy used by fish to adapt to the conditions^47^. Apart from blood glucose, biochemistry changes in the blood are also used in evaluating the health or stress condition of fish^48–50^. According to Table 2, different water levels gave no significant effect (*p* > 0.05) on the parameters of BG, TP, SGPT, BUN, and creatinine. In general, the blood biochemistry responses are still in normal conditions (no stress).

The normal values of BG, BUN, and creatinine in the fish range from 29 to 43 mg/dL^51^, 1.8–7.1 mg/dL, and 0.8–1.5^52^. Several previous studies also showed various BG between 41.17 and 246 mg/dL^42,53–55^. The TP value in this study ranged from 3.63 to 3.39 g/dL. This value was lower than that before the treatments. The TP value in this study does not show a big difference from the values of other studies that ranged from 3.90 to 5.11 g/dL^56–58^. The SGPT value in this study was 1 IU/L; it is lower than that before the treatments. Besides, this value is much lower than the values of other species, such as *A. marmorata* with 4.53–8.27 IU/L^59^, *Esox Lucius* with 4.9–11.2 IU/L, *Salmo solar* with 6 IU/L, *Salmo trutta* with 1.19–41.99 IU/L, and *Timalus timalus* with 0.59–71.98 IU/L^60^. The SGPT values are influenced by fish species^61^. BUN and creatinine are indicators for kidney organ damage. The increase of BUN indicates the declining ability of the kidneys to excrete urea in the blood. It is also in line with the creatinine; the increase of the creatinine signifies damage to the kidney organs^52^. In this study, the BUN and creatinine values were 5 mg/dL and 0.39–0.40 mg/dL, respectively. Other studies show somewhat similar results, for example *Clarias gariepinus* with 0.00–6.30 mg/dL and 0.00–2.90 mg/dL^52^, *Gymnothorax vicinus* with < 11 and 26–31 mg/dL^62^, *Notopterus notopterus* with the values of 2.20 and 0.69 mg/dL^63^.

The water level treatment had a significant effect (*p* < 0.05) on SGOT and ALP. The highest SGOT value was found in treatment A with 128.44 IU/L. Treatment C and D showed relatively similar findings; and the lowest SGOT was found in treatment B with the value of 100.11 IU/L. The SGOT value after the treatments was lower than that before the treatments. In *A. marmoratta*, with different density, the SGOT values ranged from 114 to 183 IU/L^59^. Meanwhile, the values of other species include *Esox lucius* with 252–583 IU/L, *Thymallus thymallus* with 59.98–119.97 IU/L, *Salmo trutta* with 71.98–719.85 IU/L, and *Salmo salar* with 278 IU/L^60^, *Gymnothorax vicinus* with 108 IU/L^62^, *Oreochromis niloticus* with 139–204 IU/L^64^. The highest ALP value was found in treatment C with a value of 288.89 IU/L. The lowest ALP value was found in treatment A, which was 249.33 IU/L. The values of treatment B and D were 267.44 IU/L and 225.72 IU/L, respectively. The ALP value before the treatments was 265.00 IU/L. This value showed a decrease in treatment A and an increase in other treatments as compared to that before the treatments. In another study investigating the species of *A. anguilla*, it was found that the rise of ALP value greater than 50% was due to the exposure of propenyl; the controlled ALP value was 35.46 IU/L^65^. The ALP value also varies in the *A. bicolor* eel found in different seasons with 98.6–154.7 IU/L^66^.

The hematology is a significant indicator in aquaculture as it can provide an evaluation of the health status of fish due to changes in nutrition, water quality, and disease; besides, it can be done in a non-lethal way^54,67^. The hematological in this study showed relatively similar responses in all treatments (*p* > 0.05). These responses were still in normal conditions (no stress). The normal RBCs values are in the range of 1.01–2.43 × 106 cells/mm^3^^3,10,11,20,68^. Other studies also showed an increase of the RBCs values with 1.59–1.79 × 106 cells/mm^3^ and 1.59–1.79 × 106 cells/mm^3^^3,20^. WBCs have been used in the clinical evaluation of stress and fish disease^71^. In this study, the number of WBCs showed a decline in the amount of 9.27–7.06 × 104 cells/mm^3^. However, this decline is still within the normal range. The normal SDP of fish is in the range between 3.60 and 7.58 × 104 cells/mm^3^^72^. The decrease in WBCs was also reported at the level of 8.1–5.4 × 104 cells/mm^3^^70^. The low number of WBC implies that the fish are healthy and have a good immune response^73,74^.

Hb functions as an indicator that shows the blood ability to carry oxygen^73^. The values of Hb in this study ranged from 8.45 to 8.93 g%; this shows a normal range for the eels. The normal Hb values in a fish range from 4 to 11 g%^20,42,68,75^. Meanwhile, He is the ratio between RBCs volume and total blood volume^76^. In this study, the water level treatments had a significant effect (*p* < 0.05) on the He value. The highest value was found in treatment D with 27.31%. This result was similar to that of treatment B, but different from those of treatment A and C. The lowest He value was in treatment C that was 25.20%. In general, the He value increased by 21.60% as compared to that before the treatments. In general, the He value in this study is still in the normal range. The normal He value in the eel’s blood of eel is between 26 and 36%^72,77^.

Leukocyte differentiation which includes monocytes, lymphocytes, and neutrophils is a derivative of WBCs^70^. The comparison among monocytes, neutrophils, and lymphocytes has been an excellent indicator for measuring the stress level of fish^78^. In this study, the monocytes of the initial conditions before the treatments were 25.20%. After the treatments, the monocytes increased by 10.58–14.59%. The monocytes showed a very low percentage; it is in line with^79^. The lymphocytes in the initial condition before the treatments was 61.80%. Then, it showed various results after the treatments ranging from 71.37 to 73.62%. The decrease of monocytes is due to the increase of lymphocytes produced by antibodies^80^. Similar to the lymphocytes, the neutrophils also showed the same response, in which it was low before the treatments began and increased as the treatments proceeded. Another study found that eels weighing 120 g had 100 WBCs, 34–70% lymphocytes, and 8–29% neutrophil granulocytes^81^.

The results of the histological analysis showed that the eels reared at different water levels indicated several changes of their histological structure, which is gill and skin damage (Fig. 1). Filament necrosis occurred in the gill organs for all treatments. The skin also experienced some erosion on its epidermis for all treatments. The changes in the histology structure that occurred were due to the fact that eels were in a very low water level, thus providing a moment for gills and skin to be in direct contact with air. This condition allows the uptake of air gases continuously and causes irritation to the gills and skin organs. Changes in the histology structure did not significantly affect the production performance and health status of eels in all treatments. Production performance escalated with increasing water level. SR values that reach 100% and significant growth are important indicators that the fish are in good health status. Necrosis and hyperplasia also occurred in *A. japonica* due to the exposure to mercury and infection of *Herpesvirus anguillae*^82,83^. Changes in the histology structure also occur in several vital organs due to diseases and contaminants^20,84–87^. Erosion on its epidermis occurred because the skin was in direct contact with air continuously and caused the outer part of the skin to fade. Changes in the histology structure of the skin did not have a negative effect on other parameters (production performance and physiological responses), eels showed good production performance with undisturbed health status due to treatment. Eels are strong species and able to withstand extreme conditions, this is supported by a strong and thick skin structure^88^. It is capable to protect the body surface from chemical damage and infection of microorganisms^1,84,89–91^. The results of other studies also show the same symptoms, namely the thinning or erosion of epidermal cells due to pathogenic infections^92^.

In general, the water quality parameters were still in the optimum range for all treatments. The highest temperature concentration was found in treatment (28.48 °C) and the lowest was in treatment D (27.01 °C). It was still in the normal conditions for the eel rearing. The optimal temperature range for eel rearing itself ranges from 22 to 33 °C^42,93–95^. The concentration of pH during the study ranged from 6.88 to 6.92 it was still under the normal conditions for eel rearing. The optimal pH range in the eel rearing ranges between 6 and 8^94–96^. The highest DO concentration was identified from treatment A (5.28 mg/L) and the lowest was from treatment B (4.90 mg/L), it was below the normal conditions for the rearing as the optimal rate for the eel rearing is > 3.0 mg/L^94,97,98^.

The concentration of TAN in this study ranged between 0.42 and 0.51 mg/L, which was below the normal conditions for eel rearing, the optimal value of TAN for eel rearing is < 0.25 mg/L^96^. The concentration of nitrite in this study ranged from 0.84 to1.58 mg/L, which was still under normal conditions. The ideal concentration of nitrite in the eel rearing is < 0.5 mg/L^99^, and < 0.1 mg/L^96^. Nitrite is less toxic than ammonia with a tolerance level of 0.4–0.8 mg/L^100^. The concentration of ammonia ranged from 0.0024 to 0.0038 mg/L, which is below the best conditions for eel rearing. The best range of ammonia in eel rearing is < 0.1 mg/L^94,96,101^. Alkalinity ranged between 13.42 and 16.42 mg/L. These conditions are ideal for eel rearing as the optimal alkalinity for the eel rearing is 30–500 mg/L^102,103^. The normal conditions of the water quality in this study were highly influenced by the use of a recirculation system. The recirculation system is an intensification of fish production by reusing the rearing water and processing the water to depurate it^104–106^. Water management is carried out by using filters to reduce fish culture waste and feed remains^107,108^.

Elver eels *A. bicolor bicolor* could be reared in a container arranged vertically with a recirculation system and in most cases give the same responses to different water levels. The water level of 1.5 cm provides advantage in several ways namely the least in water use and gives blood biochemistry values that are the closest to normal condition, also the best concentration of temperature and DO.

### Ethics declaration

We ensured that the experiments followed the ethical guidelines of IPB University and confirmed that all experimental protocols were approved by IPB University.

## Data Availability

The datasets generated during and/or analysed during the current study are available from the corresponding author on reasonable request.
